# Pleiotropic Contribution of *MECOM* and *AVPR1A* to Aggression and Subcortical Brain Volumes

**DOI:** 10.3389/fnbeh.2018.00061

**Published:** 2018-04-03

**Authors:** Marjolein M. J. van Donkelaar, Martine Hoogman, Irene Pappa, Henning Tiemeier, Jan K. Buitelaar, Barbara Franke, Janita Bralten

**Affiliations:** ^1^Department of Human Genetics, Radboud University Medical Center, Nijmegen, Netherlands; ^2^Donders Institute for Brain, Cognition and Behaviour, Radboud University, Nijmegen, Netherlands; ^3^Department of Child and Adolescent Psychiatry/Psychology, Erasmus Medical Center, Rotterdam, Netherlands; ^4^Department of Psychiatry, Erasmus Medical Center, Rotterdam, Netherlands; ^5^Department of Epidemiology, Erasmus Medical Center, Rotterdam, Netherlands; ^6^Department of Cognitive Neuroscience, Radboud University Medical Center, Nijmegen, Netherlands; ^7^Karakter Child and Adolescent Psychiatry, Radboud University Medical Center, Nijmegen, Netherlands; ^8^Department of Psychiatry, Radboud University Medical Center, Nijmegen, Netherlands

**Keywords:** aggression, genetics, MRI, brain imaging, neurobiology

## Abstract

Reactive and proactive subtypes of aggression have been recognized to help parse etiological heterogeneity of this complex phenotype. With a heritability of about 50%, genetic factors play a role in the development of aggressive behavior. Imaging studies implicate brain structures related to social behavior in aggression etiology, most notably the amygdala and striatum. This study aimed to gain more insight into the pathways from genetic risk factors for aggression to aggression phenotypes. To this end, we conducted genome-wide gene-based cross-trait meta-analyses of aggression with the volumes of amygdala, nucleus accumbens and caudate nucleus to identify genes influencing both aggression and aggression-related brain volumes. We used data of large-scale genome-wide association studies (GWAS) of: (a) aggressive behavior in children and adolescents (EAGLE, *N* = 18,988); and (b) Magnetic Resonance Imaging (MRI)-based volume measures of aggression-relevant subcortical brain regions (ENIGMA2, *N* = 13,171). Second, the identified genes were further investigated in a sample of healthy adults (mean age (SD) = 25.28 (4.62) years; 43% male) who had genome-wide genotyping data and questionnaire data on aggression subtypes available (Brain Imaging Genetics, BIG, *N* = 501) to study their effect on reactive and proactive subtypes of aggression. Our meta-analysis identified two genes, *MECOM* and *AVPR1A*, significantly associated with both aggression risk and nucleus accumbens *(MECOM)* and amygdala *(AVPR1A)* brain volume. Subsequent in-depth analysis of these genes in healthy adults (BIG), including sex as an interaction term in the model, revealed no significant subtype-specific gene-wide associations. Using cross-trait meta-analysis of brain measures and psychiatric phenotypes, this study generated new hypotheses about specific links between genes, the brain and behavior. Results indicate that *MECOM* and* AVPR1A* may exert an effect on aggression through mechanisms involving nucleus accumbens and amygdala volumes, respectively.

## Introduction

Aggression is a common but heterogeneous phenotype often associated with psychiatric disorders that may be harmful to others (Baron and Deborah, 1994; Miczek et al., 2002). The term covers a wide range of human behaviors, varying from verbal aggression and bullying to physical violence. Together, these behaviors have been associated with a large emotional and financial burden on society, while interventions typically still have small effects (McGuire, 2008; Bakker et al., 2017). To address aggression-related negative outcomes more successfully, a better understanding of the genes and neural mechanisms that control this behavior is essential.

Although heritability estimates differ as a function of the population and the type of aggression that is investigated, twin studies show that about 50% of the variance in aggression can be explained by genetic influences, implicating a role for genetics in the development of aggressive behavior (Tuvblad and Baker, 2011; Veroude et al., 2016). Despite this considerable heritability of aggression, the identification of specific genetic risk factors has been difficult. One factor complicating gene-finding is the largely polygenic nature of aggression. While some monogenic disorders leading to aggression phenotypes do exist (the most well-known example perhaps being Brunner syndrome; Brunner et al., 1993), multiple genetic variants, each with a small effect size, contribute to the aggression phenotype in most individuals. Because of the hypothesized polygenic model of multiple common variants with small effects underlying aggression, studies have investigated the role of these common variants by conducting association studies. Next to early candidate genetic approaches relying on *a priori* biological hypotheses, several genome-wide hypothesis-generating approaches to gene finding have now also been conducted. The main focus of candidate gene studies of aggression has been on genes related to brain neurotransmitter function, in particular to serotonergic and dopaminergic genes, and on genes related to neuroendocrine signaling, like sex-steroid receptors and stress-related circuitry (Iofrida et al., 2014; Fernàndez-Castillo and Cormand, 2016; Waltes et al., 2016). Genome-wide association studies (GWAS) of aggression have investigated a wide range of aggression related phenotypes. Interestingly, two genes showed evidence for association based on more than one GWAS, *NFKB1* and *A2BP1*. *NFKB1* encodes the nuclear factor of kappa light polypeptide gene enhancer in B-cells 1, a transcription regulator involved in axonal regeneration and degeneration. *A2BP1* (also called *RBFOX1*) encodes the RNA binding protein, fox-1 homolog (*C. elegans*) 1, a neuron-specific RNA splicing factor that regulates the expression of large genetic networks during early neuronal development (Fernàndez-Castillo and Cormand, 2016). Recently, a large-scale GWAS meta-analysis was conducted within the framework of the early genetics and lifecourse epidemiology (EAGLE) consortium, including nearly 19,000 subjects. The researchers combined GWAS data on childhood and adolescent aggression from nine population-based cohorts, and found suggestive evidence of association for a region on chromosome 2, near a gene involved in the regulation of excitatory synapse development (Pappa et al., 2015). While most other GWASs of aggression were relatively small-scaled, top-finding of these studies together with bioinformatics approaches have highlighted the importance of neurodevelopmental and synaptic plasticity genes for aggression risk (Fernàndez-Castillo and Cormand, 2016).

Investigation of the neural correlates of aggression has highlighted the involvement of several brain phenotypes in aggression. The main forms of cognitive dysfunction that have been recognized in the context of aggression are decreased empathy, an increased acute threat response and impaired decision-making. Different neural systems are thought to underlie these cognitive impairments. In short, the main neural substrates of empathic processing are thought to be the ventromedial prefrontal cortex (vmPFC) and the amygdala, while the acute threat response is mediated by the amygdala-hypothalamus-periaqueductal gray neural system, and poor decision-making in individuals with aggressive behavior has been related the striatum and vmPFC (Blair et al., 2016). Imaging studies point towards an important role for subcortical brain regions in the neurobiology of aggressive phenotypes (Siever, 2008). Of specific interest in the context of aggression are the amygdala and the striatal subregions nucleus accumbens and caudate nucleus (Blair et al., 2016). The amygdala has been strongly linked to aggression through its role in emotion processing and threat reactivity (Fusar-Poli et al., 2009; Mobbs et al., 2010). A large number of studies have reported differences in the size of the amygdala between aggressive and comparison subjects, predominantly volume reductions (Sterzer et al., 2007; Fairchild et al., 2013; Zhang et al., 2013; Pardini et al., 2014; Wallace et al., 2014; Caldwell et al., 2015; Thijssen et al., 2015; Noordermeer et al., 2016). The striatum has been associated with aggression through its central role in reward sensitivity, processing of punishment and regulation of avoidance behaviors (Finger et al., 2008, 2011; Crowley et al., 2010; White et al., 2013). Impairments in these functions are thought to be the basis of poor decision-making in individuals with aggressive behavior (Fairchild et al., 2009; Blair et al., 2016). Both volume reductions and volume increases of the striatum have been related to aggressive phenotypes, especially for the caudate nucleus and nucleus accumbens (Nosarti et al., 2005; McAlonan et al., 2007; Ducharme et al., 2011; Schiffer et al., 2011; Fairchild et al., 2013; Cha et al., 2015). Brain volume has been shown to be heritable, and the Enhancing Neuro Imaging Genetics through Meta-Analysis (ENIGMA) consortium recently conducted a GWAS meta-analysis on volumes of seven subcortical brain structures and intracranial volume, to identify genetic variants that influence brain structure (Hibar et al., 2015). Identification of such genetic variants may help to uncover mechanisms underlying neuropsychiatric disorders.

Since both aggression risk and brain volumes are heritable, one may hypothesize that part of the genes contributing to aggression neurobiology do so by influencing aggression-related brain volumes. Identification of these genes may highlight specific pathways from gene to aggressive behavior via the brain. However, research into the underlying genetic and neurobiological mechanisms of aggression is complicated by the fact that aggression is a behaviorally and etiologically complex phenomenon. Efforts have been made to recognize different subtypes of aggressive behavior, presumed to differ in their underlying neurobiology. A frequently used system divides aggression into three subtypes; proactive aggression, reactive aggression due to external provocation or threat, and reactive aggression due to internal frustration (Dodge and Coie, 1987; Raine et al., 2006; Brugman et al., 2017; Smeets et al., 2017). Proactive aggression has been related to psychopathic traits and delinquent behavior (Cima et al., 2013). In this subtype, dysfunction in neural circuitry involving (venteromedial) prefrontal and striatal areas is thought to underlie observed difficulties with decision making and reinforcement learning, while a decreased responsiveness of the amygdala to distress cues is thought to reflect deficits in emotional empathy (Blair, 2013). Reactive subtypes of aggression, on the other hand, have been associated with impulsivity, anxiety and hostile interpretation bias (Bubier and Drabick, 2009; Brugman et al., 2015). It has been suggested that reactive forms of aggression are mediated by an overly responsive amygdala-related threat response circuitry, which is dependent on regulation by cortical brain regions (Blair, 2013). Hence, different pathways to the maladaptive behavior are thought to exist. An added complication for the identification of genetic and neurobiological mechanisms underlying aggression are the marked sex-differences in aggressive behaviors. Sex-differences in aggression are pronounced with respect to prevalence and with respect to type of aggression displayed (Hill, 2002; Collett et al., 2003; Stephenson et al., 2014). For example, males are overrepresented among patients with aggression-related disorders such as conduct disorder (Hill, 2002), and are more prone to display physical aggression compared to females (Baillargeon et al., 2007). Identification of subtype- and sex-dependent genetic association may help in elucidating specific links from gene to brain to behavior.

Based on the above, the aim of the current study was two-fold. First, we sought to identify genes influencing both aggression and aggression-related brain volumes. To this end, we conducted genome-wide gene-based cross-trait meta-analyses of aggression and amygdala, nucleus accumbens and caudate nucleus volume, using GWAS meta-analysis data of two large-scale consortia (EAGLE, *N* = 18,988; ENIGMA2, *N* = 13,171). Second, we aimed to assess subtype- and sex-specificity of association for identified genes. For this, we conducted gene-wide association analyses with aggression subtypes for these genes in a population sample of healthy adults with available genome-wide genotyping and questionnaire data on aggression subtypes (Brain Imaging Genetics, BIG, *N* = 501).

## Materials and Methods

### Samples

#### EAGLE

GWAS Meta-Analysis (GWAS-MA) data on aggression were obtained from the EAGLE consortium which investigated childhood aggressive behavior using nine population-based studies with a total of 18,988 subjects (mean age = 8.44 years, SD = 4.16; Pappa et al., 2015). Different well-validated parent-report questionnaires were used to assess aggressive behavior. Depending on study sample, aggressive behavior was assessed with the aggression scale of the Childhood Behavioral Checklist (CBCL), the conduct problem scale of the Strengths and Difficulties Questionnaire (SDQ), or comparable items in general questionnaires. Scores derived from SDQ and CBCL questionnaires were shown to be highly correlated and interchangeable for the assessment of children’s behavior problems (Goodman and Scott, 1999). Genomic data were imputed to the HapMap reference panel (release 22) and comprised only samples of European ancestry. GWAS was performed for each cohort, followed by removal of single nucleotide polymorphisms (SNPs) with low minor allele frequency (<0.05) and imputation quality (RSQ < 0.3 or INFO < 0.4). Results were combined using the sample-size weighted z-score method as implemented in METAL (Willer et al., 2010), controlling for genomic inflation. Access to the summary statistics was requested through http://www.tweelingenregister.org/EAGLE. All sites involved in this study obtained approval from local research ethics committees, and written parental consent was obtained for all participants.

#### ENIGMA2

GWAS-MA data on the aggression-related subcortical volumes of nucleus accumbens, amygdala and caudate nucleus were obtained from the ENIGMA consortium. The ENIGMA consortium conducted GWAS-MA on intracranial volume (ICV) and seven subcortical brain volumes, to identify common genetic variants contributing to volume differences. They used MRI brain scans and genome-wide genotype data of 13,171 subjects of European ancestry from 28 cohorts (discovery sample). Brain scans were examined and processed at each site following a standardized protocol. Subcortical volumes had been adjusted for ICV to identify specific genetic contributions to individual volumes. Genomic data comprised only European samples and were imputed to the 1000 Genomes, v3 phase1 reference panel using MaCH for phasing and minimac for imputation (Fuchsberger et al., 2015). GWAS was performed at each site, and SNPs with an imputation score of RSQ < 0.5 and minor allele count <10 were removed. Results were combined using an inverse-variance-weighted model as implemented in the software package METAL (Willer et al., 2010), controlling for genomic inflation. Further details of the original analysis can be found in Hibar et al. (2015). Access to the summary statistics of ENIGMA was requested through the ENIGMA website^1^. All sites involved in this study obtained approval from local research ethics committees or Institutional Review Boards, and all participants gave written informed consent.

#### BIG

To assess subtype-specific association of identified genes and for mediation analysis, data from the BIG study was used. This study was conducted at the Donders Institute for Brain, Cognition and Behavior (Franke et al., 2010), and consists of self-reported healthy adults who participated in smaller-scale imaging studies at the institute. Participants gave consent to use their acquired brain data, donated saliva and performed online testing. In the current study, a sub-sample of 501 subjects with available Reactive Proactive Questionnaire (RPQ) data (Raine et al., 2006), genome-wide genotype data, and structural MRI data was used (age range 18–45 years, 215 male/286 female).

All participants were of Caucasian descent and were screened using a self-report questionnaire for the following exclusion criteria before study participation: a history of somatic disease potentially affecting the brain, current or past psychiatric or neurological disorder, medication (except hormonal contraceptives) or illicit drug use during the past 6 months, history of substance abuse, current or past alcohol dependance, pregnancy, lactation, menopause and magnetic resonance imaging contraindications (Gerritsen et al., 2012). All participants gave written informed consent, and the study was approved by the regional ethics committee (Commissie Mensgebonden Onderzoek/CMO).

### Behavioral and Genetic Measures in BIG

#### Aggression Questionnaire

The RPQ was used to assess subtypes of aggression in the BIG study (Raine et al., 2006). The RPQ is a self-report questionnaire consisting of 23 items. For each item, subjects are asked to indicate, how often they have engaged in a given type of behavior, like “had temper tantrums”. Items are rated on a three-point Likert scale (“never” = 0, “sometimes” = 1, “often” = 2). Responses were summed to yield the three factors that best described the RPQ in earlier exploratory factor analysis (Brugman et al., 2017; Smeets et al., 2017) as well as in the current sample (van Donkelaar et al., 2017): “proactive aggression” (range 0–12), “reactive aggression due to internal frustration” (range 0–9), and “reactive aggression due to external provocation or threat” (range 0–10). RPQ proactive aggression scores were dichotomized into high- and low-scoring (score ≥ 2 and score ≤ 1, respectively), because of a highly positively skewed distribution in both males and females (Supplementary Figure S1). An overview of RPQ items can be found in Supplementary Table S1.

#### Genotyping and Imputation

Genetic analyses for the BIG study were carried out at the Department of Human Genetics of the Radboud university medical center. Saliva samples were collected using Oragene kits (DNA Genotek, Kanata, Canada), and genomic DNA was extracted as specified by the manufacturer. Genome-wide genotyping was performed on three different genotyping platforms; Affymetrix Genome-Wide Human SNP Array 6.0 (Affymetrix Inc., Santa Clara, CA, USA), Infinium PsychArray-24 v1.1 BeadChip^2^, and Infinium OmniExpress-24 array^2^. Quality control steps and imputation were performed using the Ricopili Rapid Imputation Consortium Pipeline^3^. Pre-imputation quality control included pre-filtering of SNPs with call rate <0.95, filtering of individuals with a genotyping rate <0.98 or inbreeding coefficient >0.02, filtering of SNPs with a call rate <0.98 or Hardy-Weinberg *p*-value < 1e-06, removal of invariant SNPs, and removal of population ancestry outliers. SHAPEIT^4^ and IMPUTE2 (Howie et al., 2009) software were used for haplotype phasing and imputation with 1000 Genomes Phase 3.v5a reference data. For the current study, best-guess genotypes were inferred with a minimum probability threshold of 0.8. Post-imputation quality control included a strict SNP imputation quality threshold ≥0.8, removal of duplicated and related individuals (pi hat > 0.25), removal of individuals with a call rate below 95%, and removal of SNPs with a call rate below 95%, a minor allele frequency of less than 1%, or failing the Hardy-Weinberg equilibrium test at a threshold of *p* ≤ 1e-6.

### Analyses

#### Genome-Wide Gene-Based Cross-Trait Meta-Analyses

We first conducted genome-wide cross-trait meta-analyses of aggression and three different aggression-related brain volume measures (amygdala, nucleus accumbens and caudate nucleus). We used summary statistic data of two large-scale GWAS of: (1) aggressive behavior in children and adolescents (EAGLE, *N* = 18,988); and (2) MRI-based volume measures of the aggression-relevant brain regions (ENIGMA2, *N* = 13,171). First, four separate genome-wide gene-based analyses with a 50 kb flanking region around genes were performed for the summary statistic data of aggression and the volumes of amygdala, nucleus accumbens and caudate nucleus using MAGMA v1.06 (de Leeuw et al., 2015). SNPs were mapped onto genes using 1000 Genomes Phase 3 reference data followed by computation of gene *p-values* by aggregating the effect of common variants within the genes. Next, fixed-effects meta-analyses were performed of aggression with amygdala volume, aggression with caudate nucleus volume and aggression with nucleus accumbens volume, using the weighted Stouffer’s Z method as implemented in MAGMA software. Results were considered significant if they reached the Bonferroni-corrected *P*-value-threshold for testing 18,310 genes (*p* < 2.731e-6). Significant genes with stronger association *p-values* in meta-analysis, compared to the separate analyses of aggression and brain volume, were reported and selected for further investigation.

#### Gene-Wide Association Analyses for Aggression Subtypes

Gene-wide association of selected genes with three subtypes of aggression, reactive aggression due to internal frustration, reactive aggression due to external provocation or threat and proactive aggression, was assessed. One phenotypic outlier (>4 SD) was removed for all analyses. Gene-wide analyses again included a 50 kb flanking region. Three base gene analysis models are available in MAGMA, each of them sensitive to different genetic architectures: Principal Component Analysis, mean-SNP and top-SNP models. For the current analysis, a multi-model approach was used, combining the results from the base analysis models into an aggregate *p*-value. Separate analyses were run for subjects genotyped on the three different genotyping arrays. Age and four population components derived from multidimensional scaling analysis were included as covariates. Sex was included as an interaction term in the model, yielding a gene *p*-value for main and interaction effects combined (*P*_full). This was followed by meta-analysis of the full model output of the three genotyping arrays, using the weighted Stouffer’s Z method as implemented in MAGMA software (de Leeuw et al., 2015). To protect against type I error, the conventional significance threshold (0.05) was lowered to correct for multiple comparisons, testing two genes and using the effective number of independent tests (*Meff*, see Li and Ji, 2005) for the aggression subtype outcomes, calculated to be 2.5 (taking into account the correlation matrix of the three aggression measures). Hence, results were considered significant if they reached a significance threshold of 0.01. Given the strong sex effects in aggression research (Collett et al., 2003; Stephenson et al., 2014) explorative analysis of sex are reported for reasons of completeness.

## Results

### Genome-Wide Gene-Based Cross-Trait (Aggression-Brain) Meta-Analyses

The MDS1 and EVI1 complex locus gene (*MECOM*) was significantly associated with the cross-trait construct of aggression and nucleus accumbens volume (*p* = 4.94e-07), and the Vasopressin Receptor 1A gene (*AVPR1A*) showed significant association with the cross-trait construct of aggression and amygdala volume (*p* = 1.64e-06). Both associations were more significant compared to the separate analyses of aggression and the respective brain volume (Table 1).

**Table 1:** Significant results of the genome-wide cross-trait meta-analyses of aggression and aggression-related brain volumes using gene-wide association statistics.

Gene	*N* SNPs	Brain volume	*P* EAGLE aggression	*P* ENIGMA2 volume	*P* cross-trait meta-analysis*
*MECOM*	219	Nucleus accumbens	1.67e-06	2.10e-02	4.94e-07
*AVPR1A*	1132	Amygdala	3.40e-05	6.77e-03	1.64e-06

Displayed are genes showing genome-wide significant association in the cross-trait meta-analysis of aggression with an aggression-related brain volume. These genes show more significant association in meta-analysis compared to gene-wide association with aggression and brain volume phenotypes separately. *Bonferroni-corrected P-value-threshold for testing 18,310 genes: p < 2.73e-6.

### Gene-Wide Association Analyses for Aggression Subtypes

The general characteristics of the 501 participants from the BIG sample included in the aggression subtype analysis are shown in Supplementary Table S2. Gene-wide association analyses with three aggression subtypes were conducted for *AVPR1A* and *MECOM* to identify gene-behavior relationships, including sex as an interaction term in the model. Association of the *AVPR1A* gene with the score for reactive aggression due to external provocation or threat was nominally significant (*P*_full = 0.016), but did not reach the corrected significance threshold (Table 2). Explorative analyses in males and females separately showed a stronger contribution to this effect for males (*P*_males = 0.037; *P*_females = 0.517). The *MECOM* gene was not associated with any of the aggression subtypes in the population sample.

**Table 2:** Gene-wide association results for aggression subtypes in healthy adults from the BIG sample (*N* = 501).

Gene	Chr	N SNPs	Start	Stop	*P* reactive internal*	*P* reactive external*	*P* proactive*
*MECOM*	3	372	168751287	169431563	0.959	0.896	0.739
*AVPR1A*	12	1583	63486539	63597971	0.709	0.016	0.734

*P-value for main and sex interaction effect combined. Chr, Chromosome. N SNPs, number of single nucleotide polymorphisms.

## Discussion

Using cross-trait meta-analyses of gene-wide association statistics, this study identified two genes as potentially pleiotropic loci for aggression and aggression-related subcortical brain volumes. We identified *MECOM* as a gene potentially contributing to both aggression risk and nucleus accumbens volume, and we identified *AVPR1A* as a gene potentially contributing to both aggression risk and amygdala volume. Subsequently, we investigated subtype-specific and sex-dependent association of these genes in an independent sample. No associations with aggression subtypes could be confirmed in this sample, although sex-dependent association of *AVPR1A* with reactive aggression due to external provocation/threat reached the nominal significance level.

The MDS1 and EVI1 complex locus gene (*MECOM)* codes for a protein known as transcriptional regulator and oncoprotein (Yoshimi and Kurokawa, 2011). *MECOM* plays an important role in early development, with Evi1 homozygous mutant mouse embryos dying approximately 10.5 days *post coitum* showing disrupted cell proliferation and disrupted development of cardiovascular and neural systems (Hoyt et al., 1997). The association *p*-value for *MECOM* in the study of aggression improved in the cross-trait meta-analysis of this behavioral trait with nucleus accumbens volume. According to our hypothesis, this might indicate that it exerts its effect on aggression through mechanisms involving the nucleus accumbens. However, we did not observe the association of *MECOM* with aggression when investigating specific subtypes of aggression in our own, smaller sample of adults. To our knowledge, little is known about *MECOM* in relation to psychiatric behavioral phenotypes so far, and future work needs to investigate this association in more detail.

Our study provides further evidence for a role of candidate gene *AVPR1A* in aggression. The Arginine Vasopressin Receptor 1A gene (*AVPR1A*) codes for the primary receptor of AVP in the brain. AVP is a neuropeptide strongly implicated in complex social and emotional behaviors, including aggression, through a host of animal studies (Ebstein et al., 2010). Also in humans, AVP was shown to play a role in enhancing aggressive behavior. For example, evidence exists for a positive correlation between aggression and cerebro-spinal fluid AVP in humans (Coccaro et al., 1998). Additional evidence comes from genetic association studies. The original aggression GWAS-MA that we used for cross-trait meta-analysis reported gene-wide association of the *AVPR1A* gene with childhood aggression (*P* = 1.61e-03), using VEGAS gene-based analysis and correcting for 21 candidate genes tested (Pappa et al., 2015). Using the MAGMA multi-model approach, which has the advantage of yielding a more even distribution of statistical power and sensitivity for a wider range of different supposed underlying genetic architectures compared to other methods (de Leeuw et al., 2015), an even lower *p*-value was reported in the current study. The cross-trait meta-analysis of aggression and amygdala volume resulted in gene-wide genome-wide significance. Other human genetic association studies of variants in the *AVPR1A* gene reported association with anger (Moons et al., 2014), gender-specific nominally significant association with pervasive aggression (Malik et al., 2014), but no association in an early study of antisocial traits (Prichard et al., 2007). While we report nominally significant subtype-specific gene-wide association of the *AVPR1A* gene with reactive aggression due to external provocation or threat in a sample of healthy adults, this finding is not significant after correcting for multiple testing. We can speculate that association of *AVPR1A* to this subtype of aggression, which specifically measures social responses to threat and provocation by others or actions of self-defense in response to others, would be in line with existing data highlighting the importance of AVP in social context and social communication. Vasopressin signaling is thought to be an important determinant of the intensity and range of social responses displayed in different social situations (Albers, 2012). For example, AVP can alter the extent to which social stimuli are threatening, by modulating sensory information (Thompson et al., 2006). Sex-dependent effects of AVP have also been found in animal research, finding opposite effects of both vasopressin and V1a receptor blockade on aggressive behavior. For example, AVP injection increases aggression in male hamsters but decreases it in females, while injection of V1a receptor antagonists has the opposite results (Gutzler et al., 2010). This data suggests that there may be a difference between males and females in the effects of vasopressin signaling on aggression. Less is known about sex differences in V1a receptor expression. Nevertheless, research in a number of species indicates that receptor distribution might vary in a sex-dependent manner as well (reviewed in Albers, 2015). Moreover, gonadal hormones can modulate the expression of vasopressin and vasopressin receptors (e.g., Dubois-Dauphin et al., 1994; Young et al., 2000), thus partly explaining sex differences in the vasopressin system. Hence, reducing phenotypic heterogeneity and taking into account sex-related heterogeneity may facilitate the search for genes involved in the etiology of aggression.

Our cross-trait meta-analysis results indicate that amygdala volume might serve as a (proxy for related) mechanisms through which the vasopressin receptor could influence aggressive behavior, and that *MECOM* may exert its effect on aggression through mechanisms involving the nucleus accumbens. Thus, we provide specific hypotheses about shared genetic risk and generated specific hypotheses about links from gene to brain to behavior for future studies to focus on. It is often assumed in imaging genetics research that genetic risk for a neurodevelopmental disorder passes through the brain phenotype to behavior. However, another possibility is that genetic factors influencing behavior also influence the brain in a way that is independent of the behavioral phenotype of interest (Kendler and Neale, 2010). Different mediation analysis methods have been developed to explore these relationships between genetic variants, brain phenotypes and behavior. Historically, the causal steps approach has been popular in mediation analysis. It uses a set of tests of significance for each path in a causal system, although a more powerful way to determine significance of a mediated effect is bootstrapping (Hayes and Scharkow, 2013). Other methods use causal modeling to describe the direction of association between different variables, using exploratory structure learning algorithms to find conditional independencies, which makes it possible to infer parts of the structure of a structural equation model (SEM) and make predictions about causation (Sokolova et al., 2017). Only a few imaging genetics studies have investigated this issue of causality earlier. Those studies showed that only part of the brain regions showing genotype effects actually do mediate between genetics and the behavior under study (Sokolova et al., 2015; van der Meer et al., 2015), proving the importance of such multilevel investigations to elucidate the biological mechanisms, by which brain alterations may be involved in aggression etiology. Currently, available methods for making causal inferences focus on SNP-level investigations, and future studies would benefit from the development of approaches for aggregating common genetic variant data to gene- or gene-set-level in mediation frameworks.

This study has several strengths and limitations. The current study used the largest data-sets available to investigate pleiotropic genetic factors for aggression and brain volumes at gene-level. Cross-trait meta-analysis of brain measures and psychiatric phenotypes is a useful way of detecting shared genetic risk and generating new hypotheses about specific links between genes, the brain and behavior (Franke et al., 2016). We were also able to use a well-phenotyped population cohort to investigate specific subtypes of aggression. While the limitations of using saliva in this cohort as a source of DNA for genotyping may be particularly relevant for the analysis of copy number variants (Fabre et al., 2011), replication of our work using DNA isolated from other sources would further strengthen the confidence in our results.

The added value of using data from such smaller cohorts over large consortium based data lies in the possibility of in-depth phenotyping and reducing sources of heterogeneity that come with the use of pooled data-sets. Nevertheless, we did not detect significant sex- and subtype-specific associations of *MECOM* and *AVPR1A* with aggression in this sample. Possibly, we still lacked power to detect small genetic effects due to sample size, or effects might be larger in non-healthy samples. Hence, future studies are needed to further investigate the specific hypotheses about gene-brain-behavior relationships generated by the current study. As this study only investigated selected subcortical MRI measures, future work should also be extended to include cortical regions as well as connectivity measures that have been shown to play a role in aggression (Meyer-Lindenberg et al., 2006).

In summary, we identified *MECOM* and *AVPR1A* as genes contributing to aggression risk in conjunction with nucleus accumbens and amygdala brain volume, respectively. Future studies may elucidate causality of gene-brain-behavior relationships. Comprehension of sex-specific physiological pathways associated with aggression subtypes is needed to enhance our understanding of the determinants of aggression, and only by understanding the mechanisms underlying different forms of aggression will we be able to develop effective treatment approaches and minimize the social costs of aggression.

## Author Contributions

MMJD, MH, JKB, BF and JB contributed to the conception and design of the study. MMJD, IP and HT contributed to data analysis and/or interpretation. MMJD wrote the first draft of the manuscript. All authors contributed to manuscript revision, read and approved the submitted version.

## Conflict of Interest Statement

BF discloses having received educational speaking fees from Shire and Medice. JKB has been in the past 3 years a consultant to/member of advisory board of/and/or speaker for Janssen Cilag BV, Eli Lilly, Lundbeck, Shire, Roche, Novartis and Servier. He is not an employee of any of these companies, and not a stock shareholder of any of these companies. He has no other financial or material support, including expert testimony, patents, royalties. The other authors declare that the research was conducted in the absence of any commercial or financial relationships that could be construed as a potential conflict of interest.

## Footnotes

^1^http://enigma.ini.usc.edu/download-enigma-gwas-results/

^2^www.illumina.com

^3^https://sites.google.com/a/broadinstitute.org/ricopili/home

^4^https://mathgen.stats.ox.ac.uk/genetics_software/shapeit/shapeit.html
