# Supplementary material for: Pleiotropic Contribution of MECOM and AVPR1A to Aggression and Subcortical Brain Volumes
Source: Front Behav Neurosci. 2018 Apr 3;12:61. doi: 10.3389/fnbeh.2018.00061 (PMC5891600; doi:10.3389/fnbeh.2018.00061)
Supplement: Supplementary file 1 [file Table_1.docx]

Supplementary Material

**Pleiotropic contribution of *MECOM* and *AVPR1A*  to aggression and subcortical brain volumes**

**Marjolein M.J. van Donkelaar1,2, Martine Hoogman1,2, Irene Pappa3,4, Henning Tiemeier3,4,5, Jan K. Buitelaar2,6,7, Barbara Franke1,2,8^#^, Janita Bralten1,2^#*^**

**^#^** Barbara Franke and Janita Bralten share last authorship

*** Correspondence:** Corresponding Author: Janita.Bralten@radboudumc.nl

# Supplementary Figures and Tables

## Supplementary Figures

**
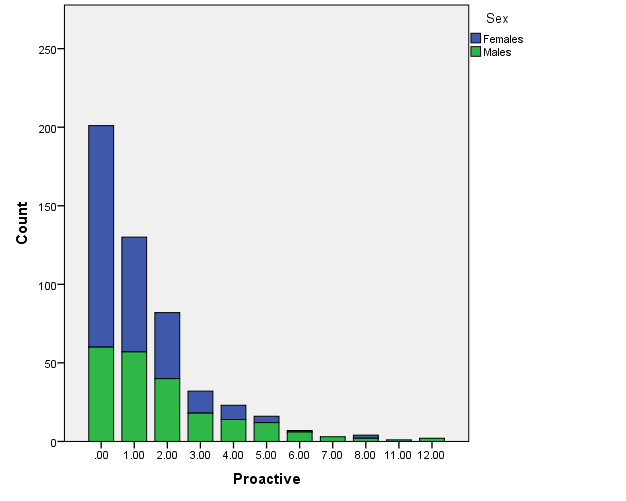
**

**Supplementary Figure 1. Distribution of RPQ proactive aggression score in males and females from our healthy adult population.**

## Supplementary Tables

**Supplementary Table 1. Reactive Proactive Questionnaire items relating to each of the aggression subtypes.**

| Item | Proactive aggression |
| --- | --- |
| 2 | Had fights with others to show who was on top |
| 4 | Taken things from other students |
| 6 | Vandalized something for fun |
| 9 | Had a gang fight to be cool |
| 10 | Hurt others to win a game |
| 12 | Used physical force to get others to do what you want |
| 15 | Used force to obtain money or things from others |
| 17 | Threatened and bullied someone |
| 18 | Made obscene phone calls for fun |
| 20 | Gotten others to gang up on someone else |
| 21 | Carried a weapon to use in a fight |
| 23 | Yelled at others so they would do things for you |
| Item | **Reactive internal frustration** |
| 1 | Yelled at others when they have annoyed you |
| 5 | Gotten angry when frustrated |
| 8 | Damaged things because you felt mad |
| 11 | Become angry or mad when you do not get your way |
| 13 | Gotten angry or mad when you lost a game |
| Item | **Reactive external provocation** |
| 3 | Reacted angrily when provoked by others |
| 7 | Had temper tantrums |
| 14 | Gotten angry when others threatened you |
| 16 | Felt better after hitting or yelling at someone |
| 19 | Hit others to defend yourself |
| 22 | Gotten angry or mad or hit others when teased |

**Supplementary Table 2. Sample characteristics Brain Imaging Genetics sample.** *RPQ proactive aggression scores were dichotomized into high- and low-scoring (score ≥ 2 and score ≤ 1, respectively), because of a highly positively skewed distribution in both males and females. Males scored significantly higher on proactive aggression than females (X^2^(1)=22.22, p <0.001; discussed in: (van Donkelaar et al., 2017)).

|  | **Total sample** | **Males** | **Females** |
| --- | --- | --- | --- |
| N | 501 | 215 | 286 |
| Mean age (SD) | 25.28 (4.62) | 24.83 (3.83) | 25.63 (5.12) |
| Mean reactive internal frustration score (SD) | 3.00 (1.81) | 3.14 (1.80) | 2.9 (1.81) |
| Mean reactive external provocation score (SD) | 2.36 (1.90) | 2.53 (1.95) | 2.23 (1.86) |
| Mean proactive score (SD) | 1.38 (1.80) | 1.93* (2.16) | 0.97* (1.33) |
